# Indents

**Published:** 1901-08-15

**Authors:** 


### INDENTS.

**☞ Brief Articles of Technical or Scientific Value will receive notice and comment. Contributions invited.**

SODIUM DIOXID AS A BLEACHING AGENT.—After the pulp canal has been properly prepared for the bleaching agent wet the inside with *boiled* distilled water, then whittle a pine stick to a long slender point with a flat side, upon this take up the dry sodium dioxid powder and carry it carefully into the cavity and deposit it on the wet walls until it gets frothy. Keep this up until the desired bleaching is obtained, usually about half an hour. Then wash the canal with hot soda water, 1 to 100, dry and pack with precipitated calcium phosphate after the suggestion of Dr. Buckley. Then fill the remainder of cavity with a properly colored cement. —Harlan, in *Dental Review*.

CLEANSING THE TEETH.—Use plenty of tooth powder in brushing the teeth, and brush the teeth thoroughly. — *Dr. J. G. Reid*. Medicated tooth powders and confections are only indicated in diseased conditions of the mucous membrane. A simple dentifrice and systematic brushing in ordinarily healthy mouths with exercise such as comes from masticating resistant foods is of great advantage.—*Dr. Harlan*. The teeth ought to be cleansed after each meal by any proceeding which will do this the most effectually. As a rule, disinfectant mouth washes are preferable to powders, as they keep the mouth sterile and prevent decay. The teeth need exercise also. — *Dr. Crouse*. People who brush their teeth twice each day, morning and evening, are likely to make each brushing more effective, as they will spend more time at it and do it more carefully.—*Dr. Wassall.*

Artistic Dentures.—Selecting harmonious shades of porcelain for artificial dentures, crowns and bridges is one of the most important features of prosthodontia, and one in which the average dentist signally fails. Efforts to imitate the harmony of shade found in the natural teeth have so uniformly failed as to make failure the rule. Manufacturers put up entire sets in a single coloring, and we do some artistic work by substituting a darker tooth occasionally to break up the excess of color harmony. But this does not suffice, as the teeth still remain conventionally of the same caste. We must imitate nature's variations if we are ever to occupy the position of artists. At present there is a demand for the best efforts the dentist can make, and just so far as he reaches this degree of artistic excellence will he receive the public's approbation and remuneration.—Dr. E. A. Royce, in *Dental Review.*

Fixing Loose Teeth in Treatment of Pyorrhea.—Silk ligatures lack permanence and cleanliness, and silver wire corrodes readily. A wire made of sixty-seven parts of silver and thirty-three parts of gold is much better. It may be used in sheets as bands or about No. 27 plate gage wire. Wire or bands assure strength and stability and the silver alloy seems to have a therapeutic advantage. If silk ligatures are used the teeth should be tied securely into proper place and then covered with

Kowarska paste to prevent the ligatures from drawing the teeth out of position or the ligatures from loosening. Apply the paste in several layers, or by using 155 grains of celluloid to 500 grains of acetone, it makes an unctious paste which can be applied in excess and will harden sufficiently in a couple of hours so that it can be trimmed to any desired form. All colors of celluloid may be had. —Harlan, in *Dental Review*.

PULP CAPPING.—Dr. M. Joseph, of Paris, recommends the following method for capping pulps: After excavating and cleansing the cavity a pledget of cotton saturated with chloroform is placed in the cavity over the pulp while the following dressing is being mixed: Mix two grains of oxid of zinc and one grain of cocain with a sufficient quantity of guaiacol to make a creamy paste. Incorporate with this paste a few fibers of asbestos to give it more body. Then remove the chloroform cotton and apply the dressing over the pulp without pressure, and cover with a disk of asbestos paper. Then fill remainder of cavity with plaster of Paris. In twenty-four hours remove this dressing and reapply the same dressing, with the exception that two grains of salol are substituted for the cocain. This second dressing is left for forty-eight hours, and is replaced by another of the same composition which is allowed to remain four or five days. It is then removed and a final dressing of oxid of zinc and guaiacol, with sufficient asbestos fiber to give it consistence, is applied and covered with sufficient plaster of Paris to retain it in place. The balance of the cavity is then filled with cement or other filling material.—W. H. Potter, in *International*.

